# A Bio-Inspired Mechanism for Learning Robot Motion From Mirrored Human Demonstrations

**DOI:** 10.3389/fnbot.2022.826410

**Published:** 2022-03-14

**Authors:** Omar Zahra, Silvia Tolu, Peng Zhou, Anqing Duan, David Navarro-Alarcon

**Affiliations:** ^1^Department of Mechanical Engineering, The Hong Kong Polytechnic University, Kowloon, Hong Kong SAR, China; ^2^Department of Electrical Engineering, Technical University of Denmark, Copenhagen, Denmark

**Keywords:** robotics, spiking neural networks, sensor-based control, visual servoing, imitation learning

## Abstract

Different learning modes and mechanisms allow faster and better acquisition of skills as widely studied in humans and many animals. Specific neurons, called mirror neurons, are activated in the same way whether an action is performed or simply observed. This suggests that observing others performing movements allows to reinforce our motor abilities. This implies the presence of a biological mechanism that allows creating models of others' movements and linking them to the self-model for achieving mirroring. Inspired by such ability, we propose to build a map of movements executed by a teaching agent and mirror the agent's state to the robot's configuration space. Hence, in this study, a neural network is proposed to integrate a motor cortex-like differential map transforming motor plans from task-space to joint-space motor commands and a static map correlating joint-spaces of the robot and a teaching agent. The differential map is developed based on spiking neural networks while the static map is built as a self-organizing map. The developed neural network allows the robot to mirror the actions performed by a human teaching agent to its own joint-space and the reaching skill is refined by the complementary examples provided. Hence, experiments are conducted to quantify the improvement achieved thanks to the proposed learning approach and control scheme.

## 1. Introduction

Robots are involved nowadays in many demanding and challenging tasks. With the aim to keep up with the pace of such demands, adaptability and novel learning techniques are essential in robots. One of the biologically inspired methods for learning is learning by demonstration or imitation, where the robot is taught by a teaching agent to execute a specific task. An issue that arises is relating the Cartesian space of both the teaching and the robot required for direct teaching from demonstrations (Argall et al., 2009; Ravichandar et al., 2020). In primates, specific neurons in several brain regions, called *mirror neurons*, are proven to trigger almost the same output while executing or observing the same task (Heyes, 2010; Cook et al., 2014). Consequently, these neurons are considered a key component in learning and refining motor skills in primates (Oztop et al., 2006; Iacoboni, 2009). A biologically inspired mechanism is introduced in this study functionally replicate the ability to learn through demonstrations. However, unlike other works in which the robot was required to just copy a certain motor skill, this work aims for the improvement of an acquired skill (i.e., target reaching) through imitation. In Iacoboni and Mazziotta (2007), mirror neurons would respond to intended tasks even with occlusions occurring indicating the sensitivity of these neurons to specific skills/actions rather than joints' movements. Hence, most studies focus on monitoring the mirroring activity in the high-order brain regions as these regions are responsible for motion planning. A study in monkeys investigated the activity in the primary motor cortex, responsible for motion transformation, after learning step-tracking while performing and observing the task (Dushanova and Donoghue, 2010). A wide set of neurons was found to attain activity while observing similar to that during acting while preserving the same preferred direction of activity, only with less amplitude. This occurs only while observing a task that was already learned by the monkey. This indicates that mirror neurons exist even in lower-order regions and may contribute to the refinement of the learned skills.

Consider a system that builds a map without any prior knowledge about body kinematics, analogous to the formation of a transformation map in the motor cortex of newborn babies (Zahra et al., 2021c). Through motor babbling, a training dataset is generated to allow building the desired map correlating the body state and the motor commands required to produce an intended motion. However, since no inverse kinematic solver or initial model of the kinematic relations is present, the motor babbling commands correspond to random movements in joint-space. For the studied case, the babbling produces waving-like motions thanks to the revolute joints utilized. It was observed that the error in the reaching actions is highly related to the collected training data of waving-like motion. This was concluded from the longer time and higher deviation from the straight target path to the target point. Hence, an auxiliary teaching mechanism is proposed to enrich the training data. One solution proposed in Kormushev et al. (2015) is a kinematic-free scheme for robot control based on generating exploratory motions to find proper motor actions. In this study, a more directed data collection is proposed where the candidate mechanism relies on learning by imitating a human agent providing more direct teaching examples. Such examples make up for the lack of proper joint coordination during motor babbling to produce motion in a straight path between numerous points in the task-space.

Surveys of different systems developed for learning from demonstrations discuss the different learning modes and challenges faced by each mode (Argall et al., 2009; Ravichandar et al., 2020). The studied case involves learning from external observations, where demonstrations are performed by a teaching agent with no sensors attached to the agent. Additionally, the policy to be learned in this case aims for low-level control of the robot in the joint space. As this case involves passive observation imitation learning, it suffers from the correspondence issue to transform the demonstration from the teacher's joint space to the robot's joint space. In Shavit et al. (2018), a dynamical system (DS) is proposed to learn from kinesthetic demonstrations. The DS is then capable of computing the desired motion to be executed in joint space to reach a target in task-space. However, no mechanism for learning from demonstrations of a teaching agent is included in the study as teaching occurs only by moving the robot links manually to execute the task (i.e., kinesthetic learning only). In Tieck et al. (2017), a spiking neural network (SNN) is introduced to reproduce the grasping motion of a hand. The data collected during a human hand grasping different objects is recorded to train the network. Then, the SNN guides the fingers of a robotic hand to grasp the objects. While the SNN reproduces the pattern of recorded movements, it does not address the case where different link lengths exist in the teaching agent/hand and the robot. Moreover, the error recorded for the joints is relatively big at the end of the training.

In this study, an SNN is developed to guide the motion of a robot through joint space motor commands in a visual servoing task. Without any prior knowledge about the robot configuration and intended direction of motion, the SNN is trained through motor babbling to provide adequate motor commands. The developed sensorimotor map is then refined by imitating the movements of a teaching agent, a human arm movement in this study, to make up for the missing knowledge about the desired movements. The teaching examples are transformed into robot coordinates through a network developed based on the self-organizing map (SOM) and Hebbian learning plasticity rule. Hence, this study contributes to the following:

- Solving the correspondence issue *via* SOMs and a biologically inspired plasticity rule.
- Improving the performance of a feedforward SNN (Zahra et al., 2021c) relying on Bayesian optimization and inhibitory interconnections.
- Validating the improvement in representation capabilities of the developed SNN *via* complementing the training data.

To the best of our knowledge, this is the first study to utilize SOMs to solve the correspondence issue for imitation learning and demonstrate the improvement in a motor cortex-like SNN architecture. The rest of this paper is structured as follows: Section 2 introduces the methodology followed for the development of the subnetworks and integration to construct the proposed network; Section 3 introduces the results obtained; Section 4 gives and discusses the conclusions of this study.

## 2. Methods

**Figure 1:**
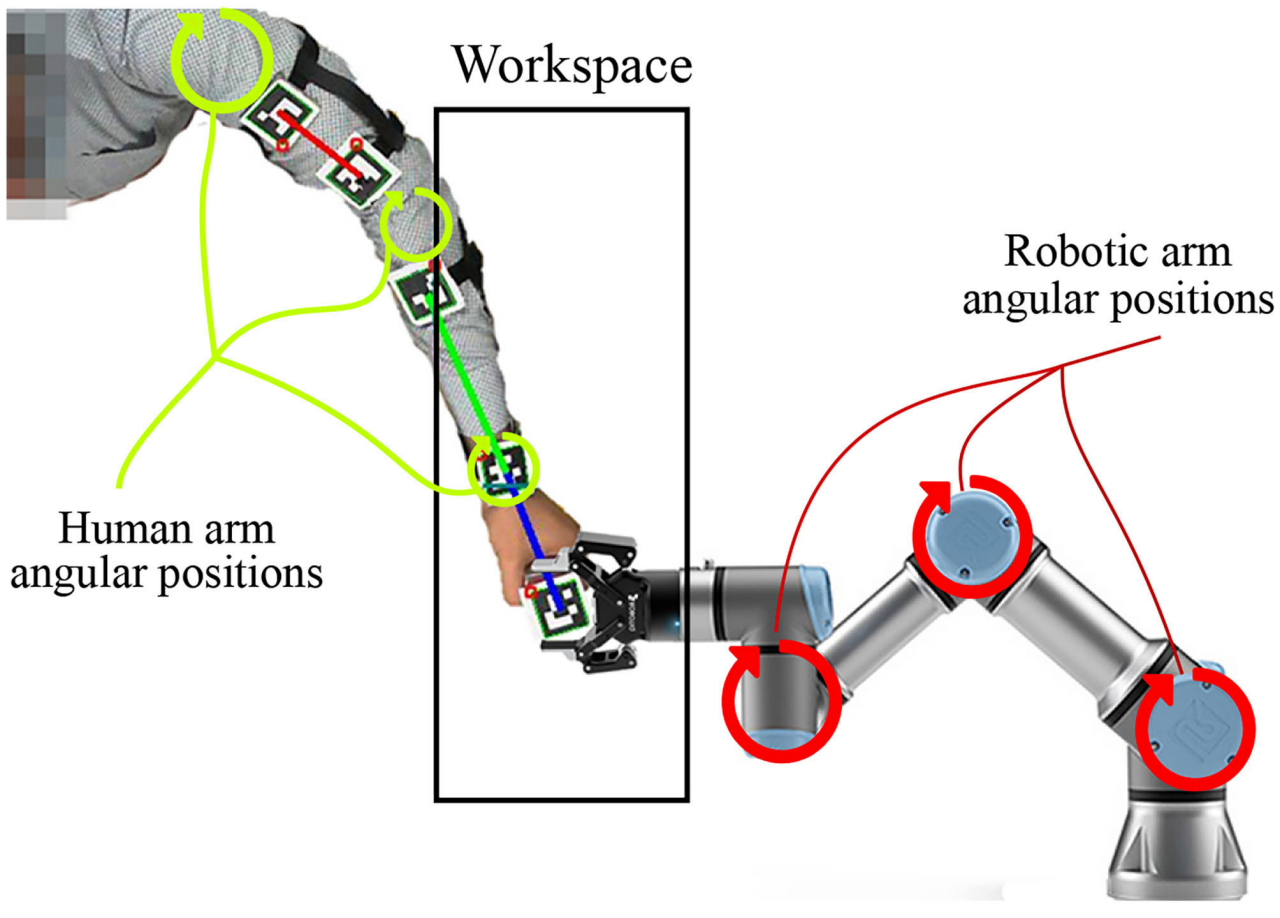
The robotic manipulator and human arm sharing the same end effector position and jointly moving during motor babbling to provide a proper training data.

While the extent of learning through imitation in humans is yet to be fully understood, this study introduces a biologically inspired mechanism that improves the quality of the target reaching skill by minimizing deviation from the intended target path. In a previous study (Zahra et al., 2021c), an SNN demonstrated the ability to learn from motor babbling and the ability to build a coarse differential map. While this map allows estimating the motor commands necessary for sensor-guided reaching of targets, the coarse estimations lead to wide deviations from the intended path. It was assumed that such deviations arise mainly due to the nature of the training set collected from waving-like motions while moving linearly in joint space. Consequently, providing better training examples, in this case, is one viable solution. In this study, the proposed mechanism acts to not only imitate actions in task space but to learn as well from the activity in joint space to refine the reaching skill. Hence, the joint space of the teaching agent (human arm in this case) is mapped to the joint space of the robotic arm. This mapping correlates the angular positions of the human arm to those of the robotic manipulator that hold the same end effector position (as shown in Figure 1). Such a correlation in angular positions allows teaching the robot and refining the reaching movements by complementing the training examples by human reaching movements after transforming into the robot's joint space (i.e., solving the correspondence issue).

### 2.1. Biologically Inspired Imitation Learning

In this study, a robotic manipulator, with *m* degrees of freedom (DoF) and a task/action space of *z* dimensions, executes a target reaching task *via* low-level joint velocity control. The kinematic relations are built based on data collected from random movements of the manipulator with no prior knowledge of configuration. Hence, the data collected as pairs of sensory readings of the joint space *JS*_*R*_ ($q_{r}\in {\mathbb{R}}^{m}$ and $u_{r}\in {\mathbb{R}}^{m}$) and the task-space *TS* ($x_{r}\in {\mathbb{R}}^{z}$ and $v_{r}\in {\mathbb{R}}^{z}$) as $m_{r}^{k}={\left\{\left\{q_{r}^{t-1,k},u_{r}^{t-1,k},x_{r}^{t-1,k},v_{r}^{t,k}\right\}\right\}}_{t=1,...,T_{k}}$, where *q*_*r*_ and *u*_*r*_ are the angular position and velocity, respectively, and *x*_*r*_ and *v*_*r*_ are the Cartesian position and velocity, respectively. *T*_*k*_ is the number of time steps taken to execute the *k*th robot reaching movement $M_{r}={\left\{\left\{m_{r}^{k}\right\}\right\}}_{k=1,...,K}$ where *K* is the total number of movements recorded.

Such random movements are executed linearly in joint space, which does not normally correspond to linear movements in the task space. Consequently, in most cases, the training data collected through robot babbling lack good examples of linear motion in Cartesian-space, which is essential to reduce the time needed for target reaching and to achieve dexterous manipulation. Thus, complementary examples are needed to enrich the training dataset. However, it is not possible to generate such examples through robot movements in absence of a mathematical model for the kinematic relations. Hence, it is adequate to provide such examples through a teacher capable of providing the desired movements. It follows that the teacher shall move across the studied *z*-dimensional work-space to provide these examples. Although the teacher can have a different number of DoFs from that of the robot, in this study, the same number of DoFs is assumed for simplicity. So, the human teacher is administered to collect the data from arm joint space *JS*_*H*_ ($q_{h}\in {\mathbb{R}}^{m}$) and the task-space *TS* ($x_{h}\in {\mathbb{R}}^{z}$) as $m_{h}^{k}={\left\{\left\{q_{h}^{t,k},x_{h}^{t,k}\right\}\right\}}_{t=1,...,T_{k}}$, where *q*_*h*_ is the angular position, and *x*_*h*_ is the Cartesian position. *T*_*k*_ is the number of time steps taken by the human arm to reach the *k*^*th*^ target. $M_{h}={\left\{\left\{m_{h}^{k}\right\}\right\}}_{k=1,...,K}$, where *K* is the total number of targets reached. Then, $M_{h}$ can be transformed *via* a separate mapping to the robot coordinates to be utilized in the learning process.

Thus, to be able to learn the policy P mapping the robot configuration to the motor actions, two modes of learning have to be adopted: (i) *learning via motor babbling* from the robot's own actions $M_{r}$, and (ii) *learning by imitating* the human teaching agent $M_{h}$ ($P\;:\;Q_{R}\;\to \;U_{R}$). The former (i.e., first mode) allows building a generalization of the differential motion achieved for specific motor commands for different configurations $P\;:\;Q_{R}\;\to \;U_{R}$ (where *q*_*r*_ ∈ *Q*_*R*_ and *u*_*r*_ ∈ *U*_*R*_). While the latter (i.e., second mode) allows refining these motions for specific desired movement paths by transforming $M_{h}$ to the robot's joint-space Ξ : *Q*_*H*_ → *Q*_*R*_ (where *q*_*h*_ ∈ *Q*_*H*_). The two learning modes are detailed in the following subsection.

### 2.2. Learning *via* Motor Babbling

**Figure 2:**
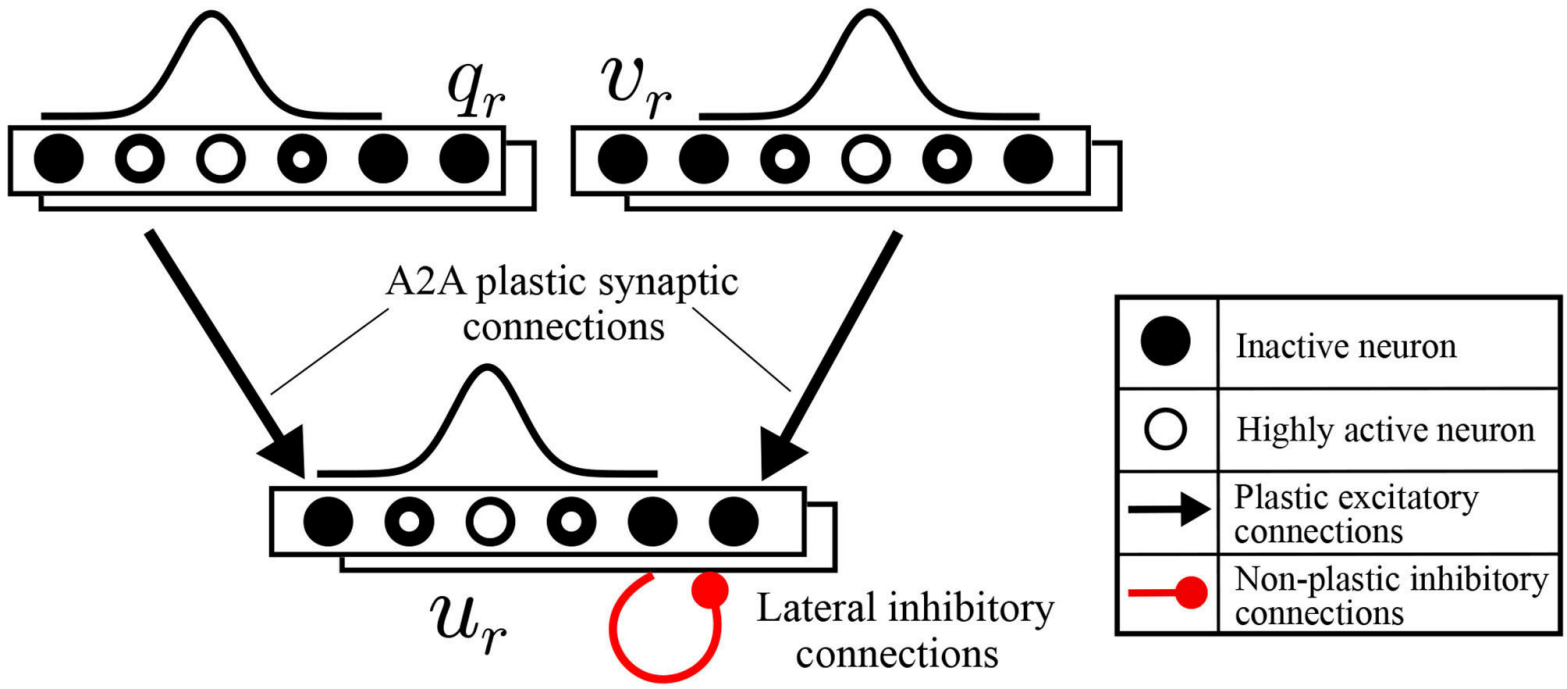
Schematic diagrams for motor cortex-like map (MCM).

**Figure 3:**
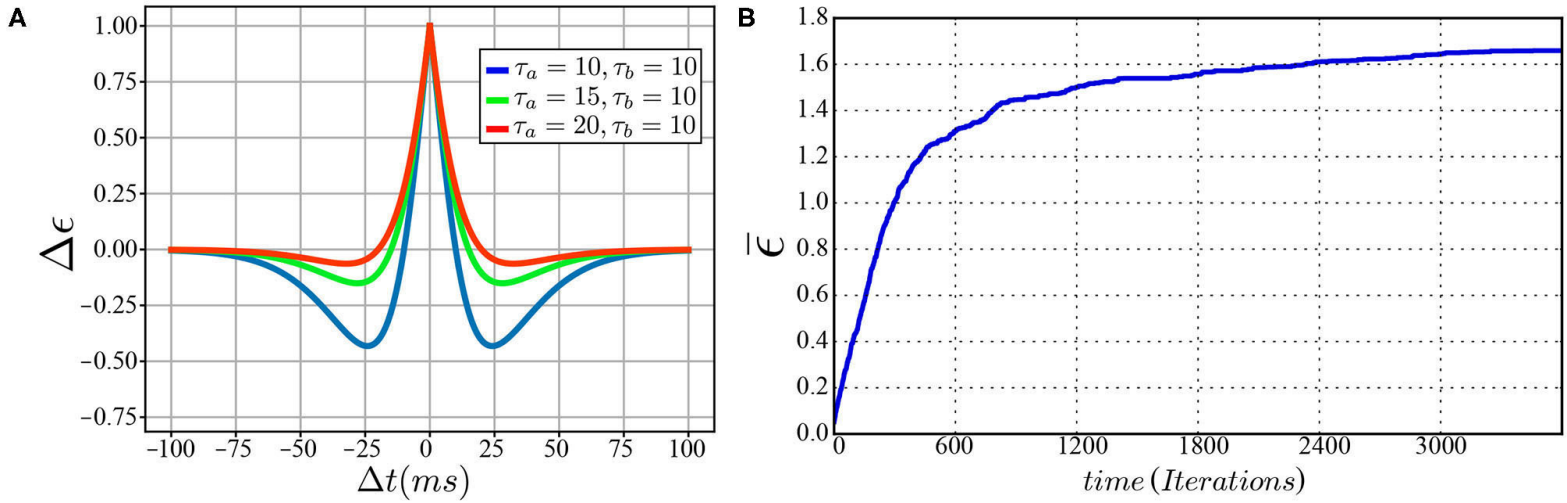
**(A)** The symmetric spike-timing-dependent plasticity (STDP) learning rule at various values for τ_*a*_ and τ_*b*_. **(B)** A plot of change in synaptic weight vs. training iterations.

To functionally emulate the motor cortex, a spiking neural network is built to transform the intended motion from task-space to motor commands. This motor cortex-like map (MCM) consists of one-dimensional arrays of neurons forming input and output layers, with each array encoding either a sensory input value or motor command output as shown in Figure 2. Input and output layers are connected through all-to-all (A2A) plastic connections obeying the symmetric spike-timing-dependent plasticity (STDP) rule (Woodin et al., 2003), shown in Figure 3A, formulated as:


(1) $$\begin{array}{l} \Delta \epsilon_{ij}=W\left(1-{\left(\frac{\Delta t}{\tau_{a}}\right)}^{2}\right)\exp\left(\frac{\left|\Delta t\right|}{\tau_{b}}\right) \end{array}$$

where Δϵ_*ij*_ is the change in the strength of synaptic connection ϵ_*ij*_ connecting the pre-synaptic neuron *i* to the post-synaptic neuron *j*. *W* defines the magnitude of the change, the ratio between τ_*a*_ and τ_*b*_ defines the window through which change (either increase or decrease) occurs, and Δ*t* is the difference between the timing of spikes at postsynaptic and presynaptic neurons. This rule is chosen as the order of spikes coming from pre and post-synaptic neurons is not relevant compared to the difference in timing of these spikes which is crucial for learning in this case. In the output layer, lateral synaptic connections allow neurons with the highest activity to suppress distant neurons for better estimations.

The neurons are modeled as Izhikevich neurons, compromising the computational cost needed and biological plausibility, demonstrated by the ability to reproduce firing patterns of neurons in various brain regions (Izhikevich, 2004). Hence, the adjustment of the parameters in the model allows for better control of the firing dynamics compared to other models. The Izhikevich neuron model is formulated as:


(2) $$\begin{array}{l} \dot{v}=f\left(v,u\right)=0.04v^{2}+5v+140-u+I \end{array}$$

(3) $$\begin{array}{l} \dot{u}=g\left(v,u\right)=a\left(bv-u\right) \end{array}$$

After a spike occurs, the membrane potential is reset as:


(4) $$\begin{array}{l} \text{if }v\geq 30\text{ mV},\text{ then }v\leftarrow c,\;u\leftarrow \left(u+d\right) \end{array}$$

where *v* is the membrane potential and *u* is the membrane recovery variable. Parameter *a* determines the time constant for recovery, *b* determines the sensitivity to fluctuations below the threshold value, *c* gives the value of the membrane potential after a spike is triggered, and *d* gives the value of the recovery variable after a spike is triggered. The term *I* represents the summation of the external currents introduced.

For the proposed network to execute the desired transformations, the information needs to be input/encoded into the network and extracted/decoded in a proper way. To be able to convert the signals from and to the network properly, the encoders (converting signals to spikes) and decoders (converting spikes to signals) are used. The input to the sensory layer (during the training and control phases) and motor layers (during the training phase only) are calculated for each neuron based on its preferred (central) value $\psi_{c}^{i}$ at which the activity of the neuron is maximum. Thus, the tuning curve for the encoders is chosen to be the Gaussian distribution. The input current to a neuron *i* for a certain input can be formulated as:


(5) $$\begin{array}{l} \kappa_{i}=A\exp\left(\frac{-{∥\psi -\psi_{c}^{i}∥}^{2}}{2\sigma^{2}}\right) \end{array}$$

where ψ is the input value, *A* is the amplitude of the input current, and σ is calculated based on the number of neurons per layer *N*_*l*_, and the range of change of the variable to be encoded from Ψ_*min*_ to Ψ_*max*_. Hence, it can be formulated as:


(6) $$\begin{array}{l} \sigma =\frac{{\text{Ψ}}_{max}-{\text{Ψ}}_{min}}{N_{l}} \end{array}$$

This leads to the contribution of the whole layer to encode a particular value (a process that can be interpreted as “population coding” Amari et al., 2003). For input neurons, κ_*i*_ is the only external current source, while for output neurons, the current is injected from both the input layer and the interinhibitory connections in the output layer. The value of *A* is chosen based on the neuron parameters and different values of activation are assigned for the sensory and motor layers as *A*_*s*_ and *A*_*m*_, respectively. The choice of *A*_*s*_ and *A*_*m*_ along with the neuron parameters allows to have a controlled firing activity and, hence, a controlled learning process. The developed network acts as a differential map to relate the robot's current configuration *q*_*r*_ and intended spatial velocity *v* with the corresponding motor command *u*_*r*_ such that:


(7) $$\begin{array}{l} u_{r}=g\left(q_{r},v\right) \end{array}$$

NeMo library allows simulating the SNN using a GeForce GTX 1080Ti GPU card with almost realtime performance (Fidjeland et al., 2009; Gamez et al., 2012). The synaptic weights are updated every algorithmic time step (one millisecond). Additionally, spikes are saved for the defined time window, through which pre-synaptic spikes are compared to a post-synaptic one to apply the STDP rule accordingly.

### 2.3. A Numerical Simulation: Proof of Concept

**Figure 4:**
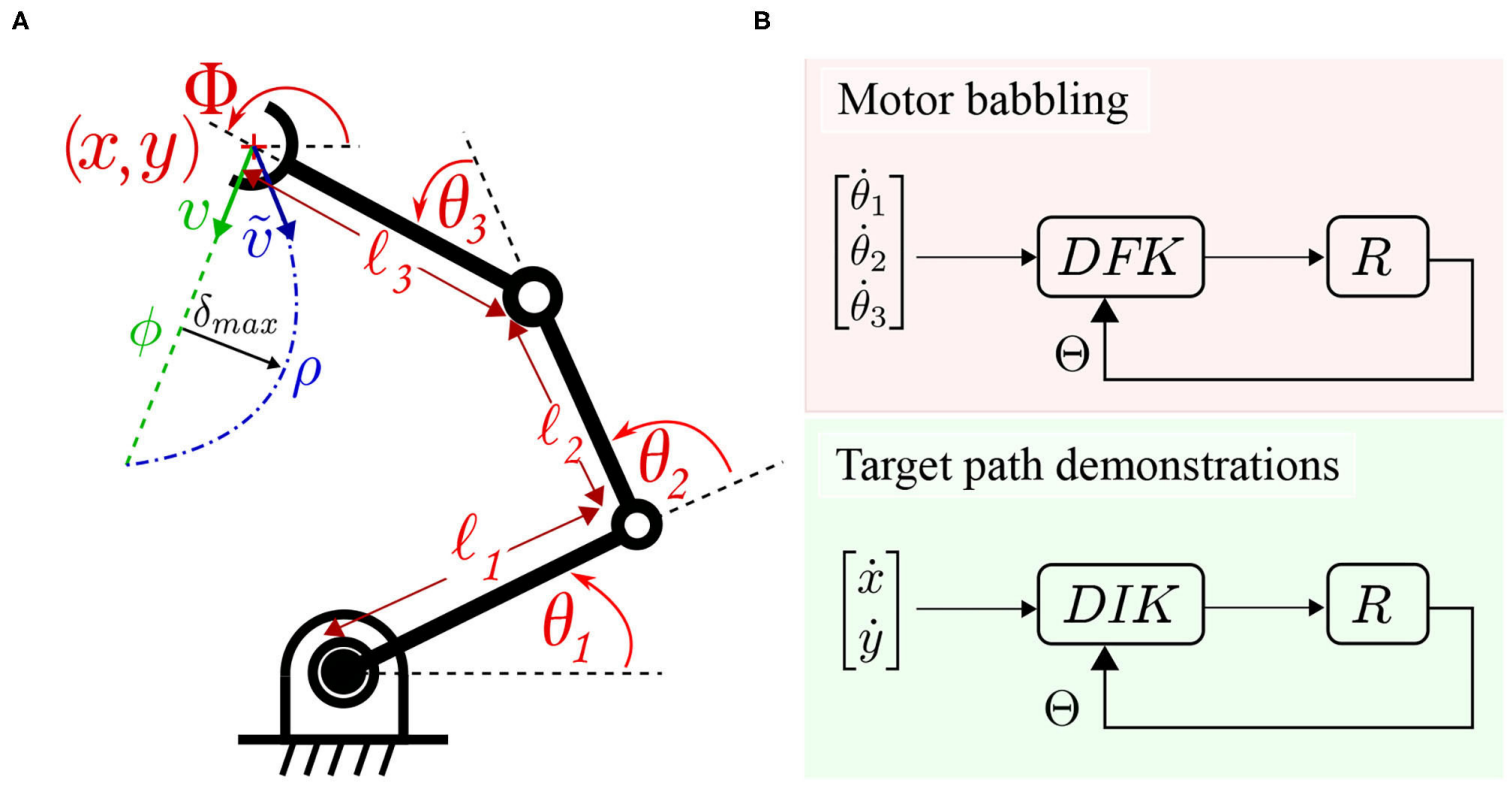
**(A)** A three link planar arm employed for numerical simulation. The robot deviates from a target path ϕ while moving from the current pose to the target pose to move through ρ instead. **(B)** The data collection schemes are illustrated. Motor babbling (in the upper panel) commands linear motion in joint space under the guidance of a differential forward kinematic *DFK* solver. Target path follower (in the lower panel) under the guidance of a differential inverse kinematic *DIK* solver.

To verify the proposed methodology before proceeding to solve the correspondence issue and real robot experiments, a simulation is designed to carry out the verification. A numerical simulation for the reaching task using a 3 link robot is set to compare the results for training using random motor babbling vs. straight path object reaching. First, the well-known forward kinematics for the robot is derived to describe the relationship between joint angles and the end effector position. Let Φ describe the orientation of the end effector, *l*_1_, *l*_2_, and *l*_3_ define the length of the 3 links starting from the base, Θ = [θ_1_, θ_2_, θ_3_] define the joints' angles as shown in Figure 4. *cθ*_*i*_ and *sθ*_*i*_ refer to cosine and sine of θ_*i*_, respectively, while *cθ*_*ij*_ refers to cosine of θ_*i*_ + θ_*j*_, and so on. The Jacobian matrix *J*(Θ) can then be derived to describe the differential relationship between the robot's joint space and task space:


(8) $$\begin{array}{l} \left[\begin{array}{c} ẋ\\ ẏ\\ \dot{\Phi } \end{array}\right]=J\left(\Theta \right)\left[\begin{array}{c} {\dot{\theta }}_{1}\\ {\dot{\theta }}_{2}\\ {\dot{\theta }}_{3} \end{array}\right] \end{array}$$

By partial differentiation of the differential forward kinematics (DFK) equations, *J*(Θ) can be obtained:


(9) $$\begin{array}{l} J\left(\Theta \right)=\left[\begin{array}{ccc} -l_{1}s\theta_{1}-l_{2}s\theta_{12}-l_{3}s\theta_{123} & -l_{2}s\theta_{12}-l_{3}s\theta_{123} & -l_{3}s\theta_{123}\\ l_{1}c\theta_{1}+l_{2}c\theta_{12}+l_{3}c\theta_{123} & l_{2}c\theta_{12}+l_{3}c\theta_{123} & l_{3}c\theta_{123}\\ 1 & 1 & 1 \end{array}\right] \end{array}$$

To collect motor babbling data, the robot moves linearly in the joint space by generating target joint angles Θ^*^ within the studied space, and commanding the robot to move to these joint angles. The joint velocities $\dot{\Theta }$ are set based on the formula:


(10) $$\begin{array}{l} \dot{\Theta }=C_{\theta }\frac{e_{\theta }}{∥e_{\theta }∥} \end{array}$$

where *C*_θ_ is a scaling gain and *e*_θ_ is the error/difference between the current joint position Θ and Θ^*^.

Then, based on Equation (8), a differential inverse kinematic solver (DIK) can be built to guide the robot's motion based on the inverse Jacobian matrix *J*^#^(Θ).

This allows moving the simulated robot in straight and curved paths by solving for the desired motor commands $\dot{\Theta }$ to move in a desired direction inside the defined workspace. This allows bypassing the correspondence problem and directly verify the efficacy of the main concepts upon which this work is built. Hence, both the collected datasets are used to train the MCM network to demonstrate the improvement achieved in this case, as discussed in the following section.

### 2.4. Learning by Imitating

**Figure 5:**
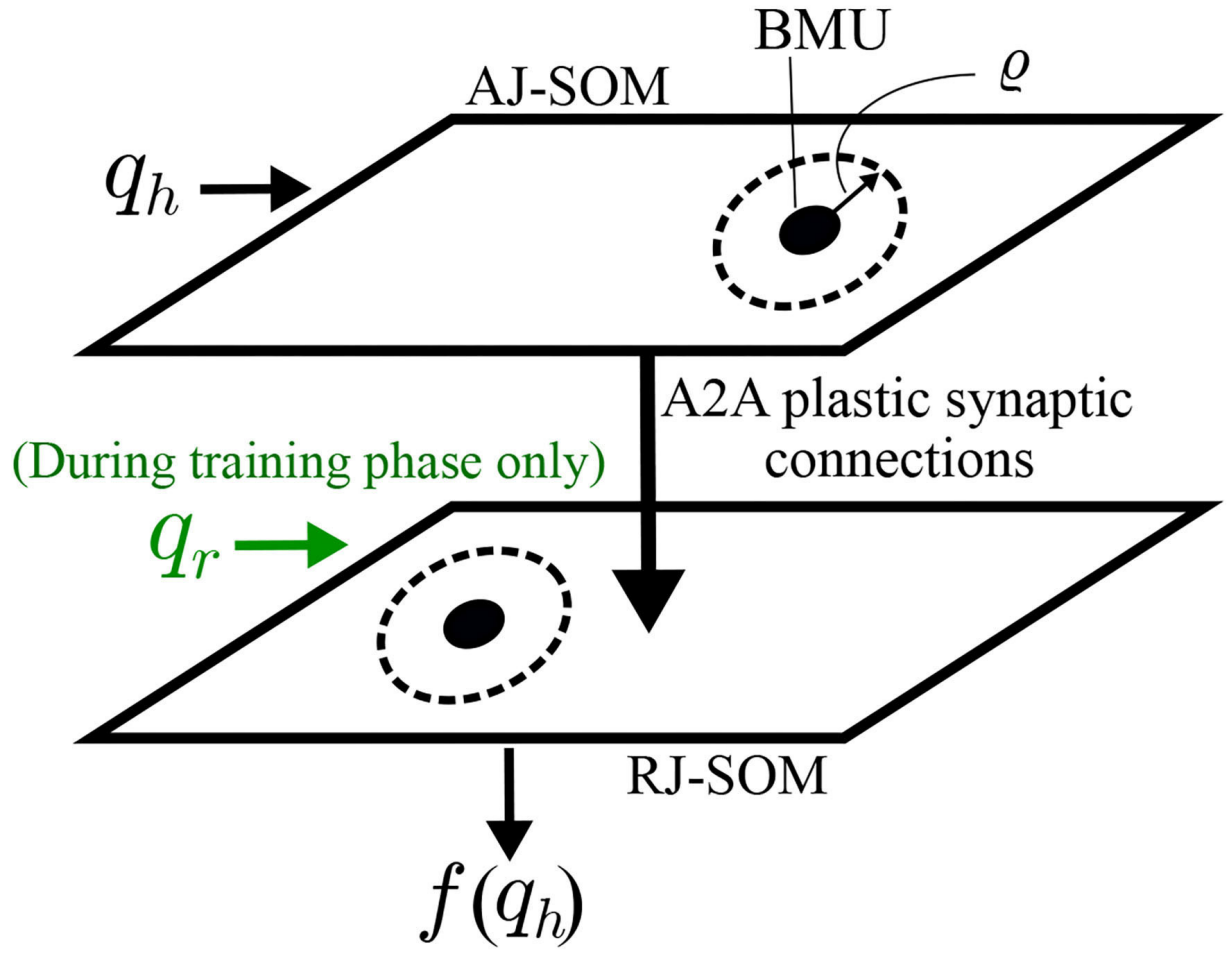
Schematic diagrams for SOMs connected through Oja-Hebbian plastic synapses. This architecture allows correlating the joint spaces of the human arm and robot arm. During the training phase, BMUs (in AJ-SOM and RJ-SOM) from both maps that fire together are more likely to have an increase in strength of the connecting synapses. Consequently, during the control phase, if the same BMU in AJ-SOM becomes active, the corresponding node in RJ-SOM becomes active as well.

To be able to imitate the human teaching agent, it is essential to solving the correspondence issue by transforming the data collected from the agent to the corresponding robot state. Thus, in the studied case, correlation of the joint spaces of both the robot and the teacher at the same position in the task space is carried out. Firstly, a representation of each of the correlated joint spaces is built using a self-organizing map (SOM) to allow for dimensionality reduction as shown in Figure 5. SOM is built upon the rules of competition, cooperation, and adaptation.

**Competition:** With each node/neuron *k* associated with a position/weight vector ω_*k*_, the nodes/neurons compete among each other by comparing the weights to that of an introduced data sample *q*. The winning node, known as Best Matching Unit *BMU*, is chosen to be with the least Euclidean distance between ω_*k*_ and *q*, such that *i* = arg min_*k*_ ∥ω_*k*_ − *q*∥, where *i* denotes the index of the BMU. **Adaptation:** The weights vector of the BMU ω_*i*_ is then updated to give a better representation of the input vector *q*. **Cooperation:** While the nodes compete to be chosen to represent an input vector, the nodes within the neighborhood of the BMU are updated as well in the adaptation phase, formulated as:


(11) $$\begin{array}{l} \omega_{j}\left(t+1\right)=\omega_{j}\left(t\right)+\lambda \left(t\right)\eta_{ji}\left(t\right)\left(q-\omega_{j}\left(t\right)\right) \end{array}$$

(12) $$\begin{array}{l} \lambda_{ji}\left(t\right)=\exp\left(\frac{-{∥p_{j}-p_{i}∥}^{2}}{2\varrho^{2}\left(t\right)}\right) \end{array}$$

where *p*_*j*_ and *p*_*i*_ are the positions of the *i*th and *j*th nodes within the SOM lattice, λ is the learning rate, η_*ji*_ is the neighborhood function, and ϱ is the neighborhood radius. Values of the learning rate and neighborhood radius are defined initially at ϱ_0_ and η_0_, respectively. As the training proceeds for *T*_*d*_, the learning rate and neighborhood radius decay such that:


(13) $$\begin{array}{l} \varrho \left(t\right)=\varrho_{0}\exp\left(\frac{-t}{T_{d}}\right),\eta \left(t\right)=\eta_{0}\exp\left(\frac{-t}{T_{d}}\right) \end{array}$$

However, one drawback of the basic SOM mentioned in the literature is the tendency to have higher approximation errors at the map boundaries (Kohonen, 2013). A model of the SOM with a varying density of nodes across the map is chosen for this study (Zahra and Navarro-Alarcon, 2019). As the output of the SOM depends on the activity of the neighborhood nodes, this model allows preserving the quality of the mapping by attracting more nodes closer to the map borders to ensure the presence of enough nodes in the neighborhood for accurate estimations. Thus, the neighborhood function differs from that of the standard SOM. A coefficient is defined for *node density* φ computed as:


(14) $$\begin{array}{l} \varphi =\exp\left(-\underset{j\in \Pi }{\sum }{∥w_{i}-w_{j}∥}^{2}\right) \end{array}$$

where Π is the local neighborhood around the node. φ allows to quantitatively find the nodes with less number of nodes in the neighborhood, and hence, more nodes shall be attracted to their proximity. Thus, the neighborhood function can be redefined to allow varying the density across the map based on φ:


(15) $$\begin{array}{l} \eta \left(t\right)={\left(\frac{t}{\varphi T_{d}}\right)}^{4}\exp\left(\frac{-t}{\varrho^{2}\left(t\right)T_{d}}\right) \end{array}$$

In our varying density SOM, the nodes within the neighborhood cooperate to give better estimations of the output. Thus, the cooperation extends as well after the training phase thanks to the varying density structure. *AJ-SOM* and *RJ-SOM* provide a representation for human arm joint-space *JS*_*H*_ and robot arm joint-space *JS*_*R*_, respectively. Each SOM is fed with data collected while holding a correspondence between *JS*_*H*_ and *JS*_*R*_, where the training examples are collected while moving in the shared workspace as shown in Figure 1. The SOMs are trained for several iterations until reaching the target accuracy of encoding for both spaces. Then, the SOMs are connected through Oja-Hebbian synapses and modulated by introducing corresponding samples to both SOMs. The activity α_*j*_ of a node *j* for an input vector *q* is then decided based on the following equation:


(16) $$\begin{array}{l} \alpha_{j}\left(t\right)=\exp\left(\frac{-{∥w_{j}\left(t\right)-q∥}^{2}}{\varrho^{2}\left(t\right)}\right) \end{array}$$

The synaptic strength is then updated based on the activity of both pre-synaptic *i* and post-synaptic *j* neurons:


(17) $$\begin{array}{l} \Omega_{ij}\left(t+1\right)=\Omega_{ij}\left(t\right)+\zeta \left(\alpha_{i}\alpha_{j}-\beta \Omega_{ij}\left(t\right)\alpha_{j}^{2}\right) \end{array}$$

(18) $$\begin{array}{l} \beta \left(t\right)=\beta_{0}\exp\left(\frac{T_{d}-t}{T_{d}}\right),\zeta \left(t\right)=\zeta_{0}\exp\left(\frac{T_{d}-t}{T_{d}}\right) \end{array}$$

where Ω_*ij*_ denotes the strength of the synaptic connection from node *i* to node *j*. The terms β and ζ are defined to adjust the learning process by adjusting the β_0_ and ζ_0_ coefficients.

**Figure 6:**
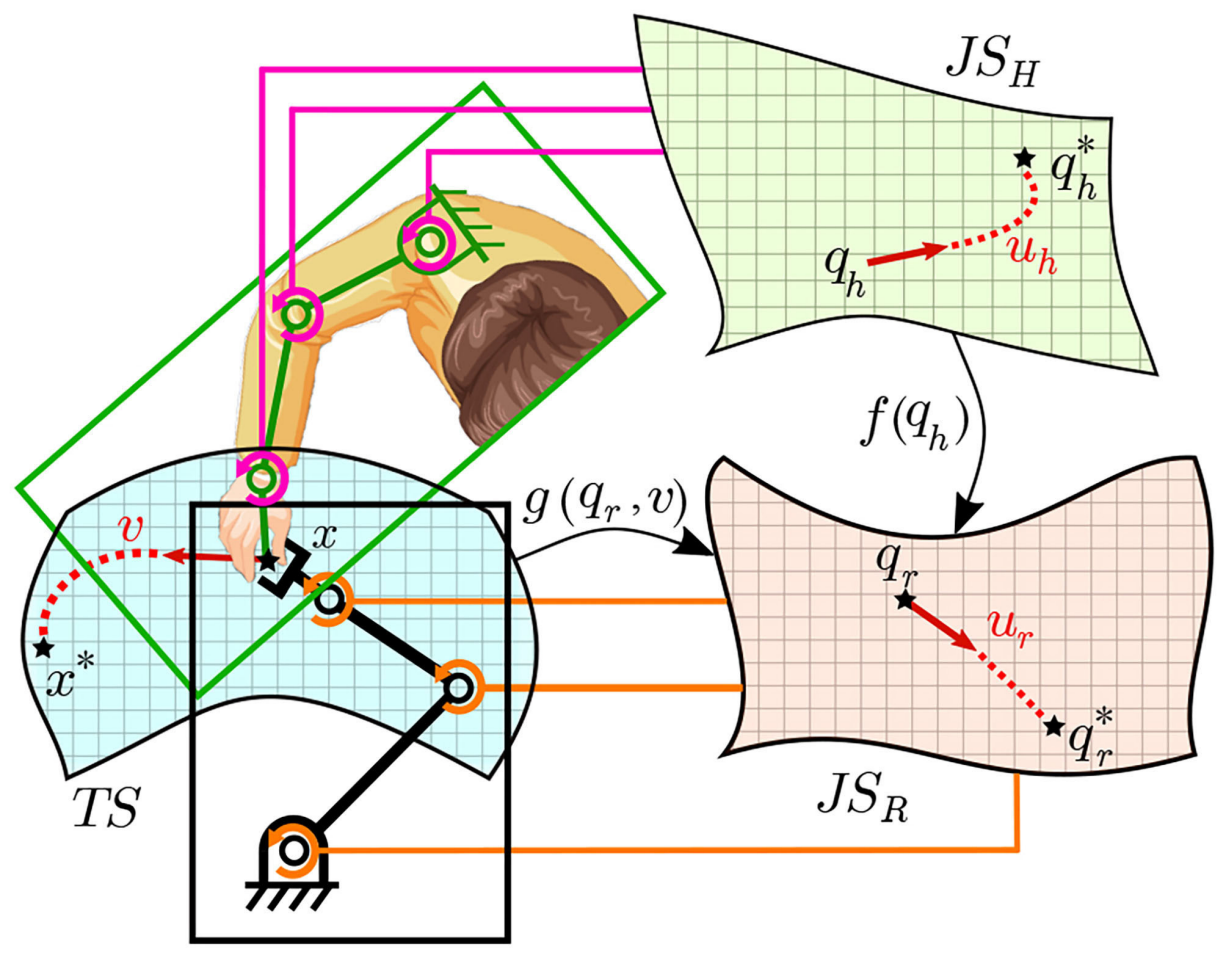
A schematic diagram of the correspondence of the human and robot joint spaces along with the task space *TS*. The data collected from the human and robot together allows building correlation (i.e., *f*(*q*_*h*_) between *JS*_*H*_ and *JS*_*R*_). This allows generating more examples to train the map *g*(*q*_*r*_, *v*) correlating *TS* to *JS*_*R*_ by transforming examples conducted by the human arm (in *TS*) into *JS*_*R*_.

**Figure 7:**
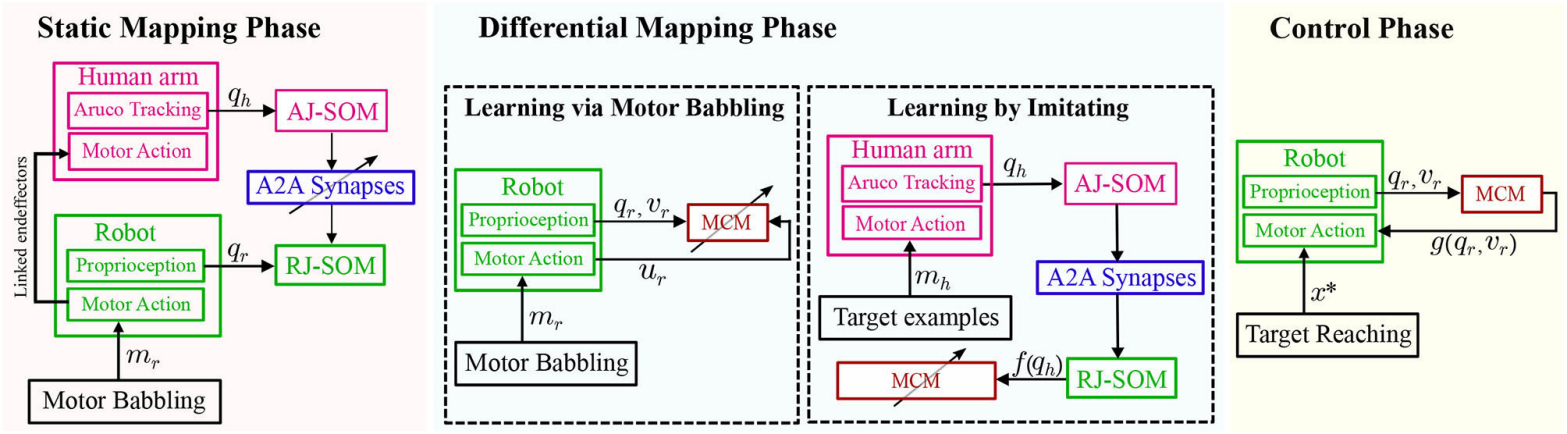
A schematic diagram of the full system including both the static (SOMs network) and differential(MCM network) maps formed. Blocks with an inclined arrow passing through are the ones where learning/adaptation occurs during each phase.

This allows for building a static mapping between *JS*_*H*_ and *JS*_*R*_ such that:


(19) $$\begin{array}{l} q_{r}=f\left(q_{h}\right) \end{array}$$

where *f* is the map formed by the described network which allows approximating the value of *q*_*r*_ corresponding to a certain *q*_*h*_ value to give the same end effector position *x* for both the human and robot agents as shown in Figure 6. Thus, the differential mapping occurs first relying on self-generated motor babbling data, followed by learning through mirrored data relying on the static map *f* built earlier, as shown in Figure 7. The working space and joint space are chosen to minimize the occurrence of redundant states.

The formed map allows the transformation of the reaching movements demonstrated by the human agent from *JS*_*H*_ to *JS*_*R*_. The angular positions of both agents, the end effector position along the timestamp are recorded while babbling at a frequency of 100 Hz, which is then downsampled to 30 Hz to allow for a significant change between the recorded subsequent points.

### 2.5. Optimizing the Hyperparameters

In this study, the hyperparameters (Γ) of MCM are optimized using Bayesian optimization with the regression model as an adaptive form of tree Parzen estimator (ATPE) (Arsenault, 2018) and the acquisition function as expected improvement (EI). The optimal values for the hyperparameters (γ^*^) are sought through minimizing an objective function *l*(Γ), given by:


(20) $$\begin{array}{l} \gamma^{*}=\underset{\gamma \in \Gamma }{\text{arg min }}l\left(\gamma \right) \end{array}$$

A probabilistic regression model gives an approximation of the objective function, defined as A=P(S|Γ) to map Γ hyperparameters to the likelihood of a score S for the chosen objective function *l*.

The *Parzen estimator* PE is a kernel-density estimator that relies on a group of continuous distributions/kernels to model some function. TPE is formulated as:


(21) $$\begin{array}{l} P\left(\Gamma \right)=\frac{1}{N_{k}\xi }\overset{N_{k}}{\underset{j=1}{\sum }}K\left(\frac{\Gamma -\Gamma_{j}}{\xi }\right) \end{array}$$

where *N*_*k*_ defines the number of the approximation kernels used, ξ is the kernel's bandwidth, and *K* is defined as a Gaussian kernel. U and D are modeled to promote hyperparameters with a higher likelihood to return lower values for the objective functions for the following observations.

The EI (Bergstra et al., 2011) can be formulated as:


(22) $$\begin{array}{l} EI_{S_{i}^{*}}\left(\Gamma_{i}\right)=\int_{-\infty }^{S_{i}^{*}}\left(S_{i}^{*}-S_{i}\right)P\left(S_{i}|\Gamma_{i}\right)dS_{i} \end{array}$$

The Bayes rule is applied to replace the posterior P(S|Γ) by P(Γ|S) instead for TPE before substituting in Equation (22) (Bergstra et al., 2011) to formulate EI as:


(23) $$\begin{array}{l} EI_{S_{i}^{*}}\left(\Gamma_{i}\right)=\frac{\mu S_{i}^{*}D\left(\Gamma_{i}\right)-D\left(\Gamma_{i}\right)\int_{-\infty }^{S_{i}^{*}}P\left(S_{i}\right)dS_{i}}{\mu D\left(\Gamma_{i}\right)+\left(1-\mu \right)U\left(\Gamma_{i}\right)} \end{array}$$

(24) $$\begin{array}{l} EI_{S_{i}^{*}}\left(\Gamma_{i}\right)\propto {\left(\mu +\frac{U\left(\Gamma_{i}\right)}{D\left(\Gamma_{i}\right)}\left(1-\mu \right)\right)}^{-1} \end{array}$$

Hence, it can be concluded that EI maximizes the ratio $D\left(\Gamma_{i}\right)/U\left(\Gamma_{i}\right)$ to provide better candidates for the search process while maintaining a balance between exploitation and exploration. The reference value of $S_{i}^{*}$ is decided by the value set for the ratio $P\left(S_{i}<S_{i}^{*}\right)=\mu$.

The time complexity for TPE is less than other BO methods (such as Gaussian Process). However, interaction among the hyperparameters is not modeled in TPE. This drawback is addressed in ATPE by concluding from Spearman correlation (Zar, 2005) of the studied hyperparameters the best parameters to tune to explore the search space efficiently. ATPE suggests empirical formulas, taking into account the search spaces' cardinality, to give optimal values of μ and the number of candidates needed by the acquisition function to generate a candidate optimal solution (Bergstra et al., 2011).

For the optimization process, the objective function is set to minimize the difference between the target *v* and the actual *ṽ* spatial velocity such that:


(25) $$\begin{array}{l} l\left(\gamma \right)=\left|\arccos\left(\frac{\vec{ṽ}\cdot \vec{v}}{∥\vec{ṽ}∥∥\vec{v}∥}\right)\right| \end{array}$$

where minimizing the value returned by the objective *l*(γ) ensures minimizing the error in estimations and hence reducing the deviation from the reference path while reaching a target. The search space for the optimization includes 15 parameters for both the neuronal units and synaptic connections. For the chosen Izhikevich neuron model, 4 parameters (*a, b, c*, and *d*) are defined for units in each layer and the parameters *A*_*s*_ and *A*_*m*_ define the amplitude of the input current to the sensory neurons and motor neurons, respectively. The other 5 parameters define the synaptic properties for the chosen spike timing-dependent plasticity (STDP) learning rule including the learning rate *W*, maximum *C*_*E*_, and minimum *C*_*I*_ synaptic weights, τ_*a*_ and τ_*b*_.

To train the SNN, examples from both the saved direct motor babbling trails and imitatory transformed trails are introduced. The motor babbling trails allow the SNN to develop the initial mapping for direct transformations, while the imitatory trails provide a complementary dataset of transformed demonstrations. Hence, the intended motion paths are demonstrated through the human teaching agent to aid in refining the formed map.

## 3. Results

### 3.1. Numerical Simulation Results

To test and quantify the improvement achieved by complementing the datasets with direct examples to reproduce these examples, the simulation, described in subsection 2.3, is employed to test moving in curved and straight target paths. With the length of the three links set as 30, 30, and 20 cm from base to end effector, the range of joint angles are set for the base, shoulder, and wrist joints as [0°, 30°], [20°, 50°], and [−10°, 30°], respectively. To assess the quality of the robot motion, the maximum deviation of the end effector from the intended path and the ability to reach the target is the chosen metrics. The intended path, denoted as ϕ, is divided into equidistant 1,000 points and the actual path, denoted as ρ, is divided similarly into 1,000 points. To check the deviation of each point ρ_*i*_ from the target path, the Euclidean distance to each point ϕ_*j*_ shall be calculated and compared to define the deviation δ_*i*_ as the least distance measured at the point ρ_*i*_, such that:


(26) $$\begin{array}{l} \delta_{i}=\underset{j}{\min}∥\rho_{i}-\phi_{j}{∥}^{2} \end{array}$$

Thus, the maximum deviation δ_*max*_ for the whole path ρ is the maximum distance measured for all of its points, hence:


(27) $$\begin{array}{l} \delta_{max}=\underset{i}{\max}\left(\delta_{i}\right) \end{array}$$

Moreover, the servoing process is considered successful if the arm reaches within a threshold of 1 mm away from the target. The data for moving in a straight line is generated by assigning a target to the robot and, consequently, a vector is concluded from the current position to the target position. By substituting for the current joint angles in *J*^#^(Θ), the joint velocities necessary to move in a straight line are calculated. The data for moving in curved paths are generated by assigning random joint angles and moving linearly in the defined joint space. This is equivalent to kinesthetic learning (*KL*) by guiding the robot movement manually. The results obtained can be summarized in Table 1 for both ϕ defined as linear or curved target paths. This concludes the feasibility and amount of improvement expected upon introducing appropriate training data to the *MCM* network. The change in weight of all excitatory synapses $\bar{\epsilon }$ is plotted against the training iterations in Figure 3B to show the learning progress. Among these connections, only 12.5% of the synaptic weights undergo change with an SD equal to 1.35 at the end of the training phase. In future studies, pruning of the inactive synapses would be included to reduce the computational cost without affecting the network's current learning capabilities.

**Table 1:** Simulation results.

**Maximum deviation**		
	**Mean (*mm*)**	**Successful trials (out of 10)**
linear w/o *KL*	53.7	6
linear with *KL*	30.1	10
curved w/o *KL*	40.6	8
curved with *KL*	15.5	10

### 3.2. Robot Setup

The human and robot agents are arranged in an adequate setup, as illustrated in Figure 1, to share the same end effector position and move jointly in the defined workspace while the robot executes the random motor babbling. The motion, in this case, is planar utilizing 3 degrees of freedom (DOF) for the agents. By visual inspection, the human agent stops the motion of the robot when the end effector moves out of the defined workspace or forces a configuration that can not be maintained by the human agent. The human arm is tracked using five aruco markers to be able to extract the angular position of each of the shoulder, elbow, and wrist joints. The posture of the human agent is maintained while collecting the data to fix a reference pose for the base coordinates of the agent.

**Figure 8:**
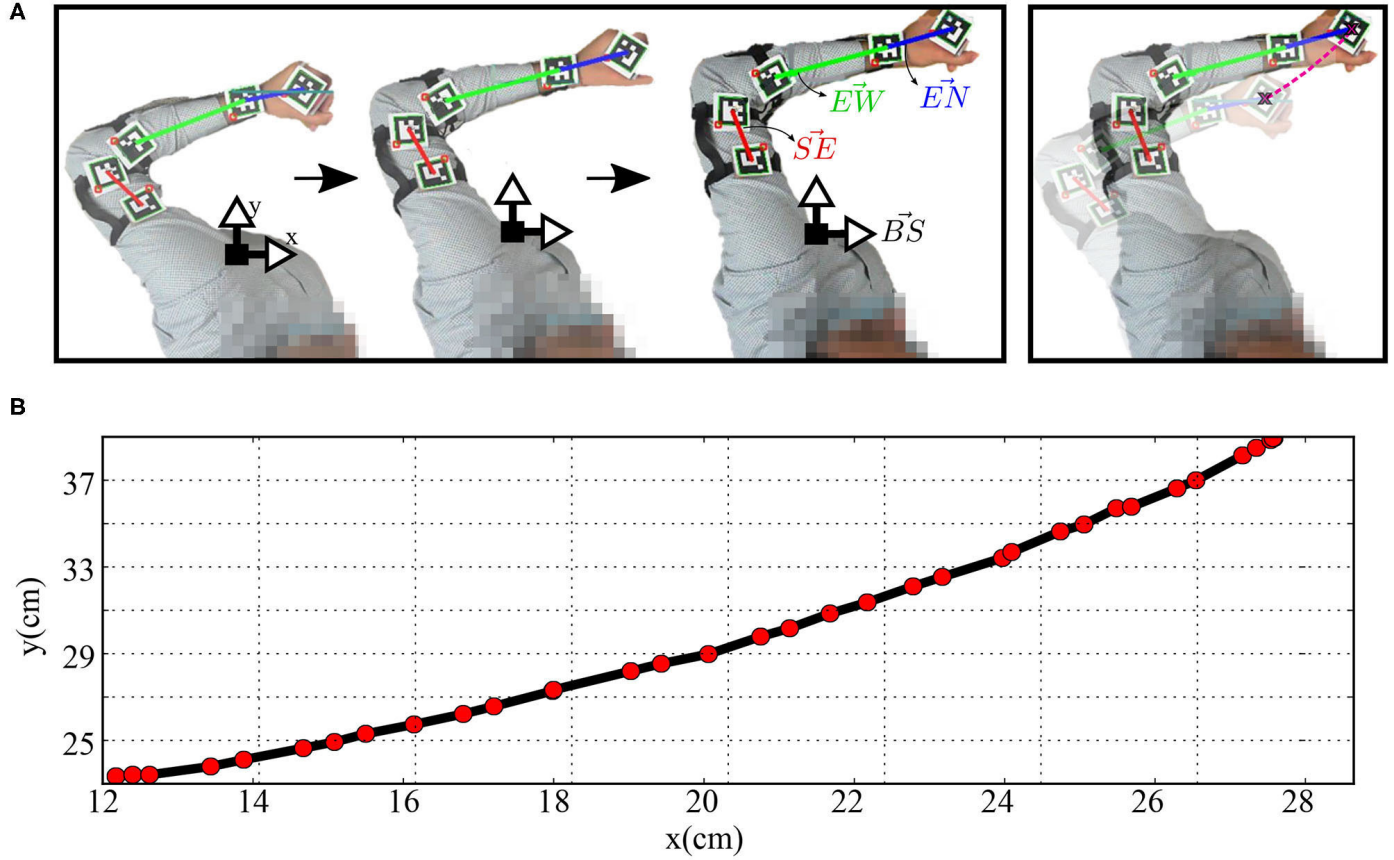
**(A)** The human arm while moving in straight paths and **(B)** the plot of the Cartesian position of the hand-held object while moving.

Two aruco markers are fixed on the arm, two markers fixed on the forearm, and one fixed on the wrist, as shown in Figure 8. Four vectors are defined to calculate the angular position *q*_*h*_; $\vec{BS}$ extends from the base coordinates and normal to the body, $\vec{SE}$ extends from the first marker to the second one (i.e., along the arm from the shoulder to the elbow), $\vec{EW}$ extends from the third marker to the fourth (i.e., along the forearm from the elbow to the wrist), and $\vec{EN}$ extends from the wrist to the end effector. The angular position $q_{h}=\left[\theta_{s}^{h},\theta_{e}^{h},\theta_{w}^{h}\right]$ can then be calculated as:


(28) $$\begin{array}{l} \theta_{s}^{h}=\arccos\left(\frac{\vec{BS}\cdot \vec{SE}}{∥\vec{BS}∥∥\vec{SE}∥}\right) \end{array}$$

(29) $$\begin{array}{l} \theta_{e}^{h}=\arccos\left(\frac{\vec{SE}\cdot \vec{EW}}{∥\vec{SE}∥∥\vec{EW}∥}\right) \end{array}$$

(30) $$\begin{array}{l} \theta_{w}^{h}=\arccos\left(\frac{\vec{EW}\cdot \vec{EN}}{∥\vec{EW}∥∥\vec{EN}∥}\right) \end{array}$$

The joint encoders provide the angular position of the robotic joints $q_{r}=\left[\theta_{s}^{r},\theta_{e}^{r},\theta_{w}^{r}\right]$. The data collected from human and robot joint spaces are used to train the AJ and RJ SOMs and train the synaptic linkage between them. This linkage allows solving the correspondence issue to provide the complimentary examples by transforming the motion executed by the teacher into the robot's joint space representation to refine the training process in the *MCM* network. After the training process ends, the end effectors of the teacher and the robot are detached to test the performance of the robot in executing the servoing task as demonstrated. The performance metrics are introduced in the next subsections.

### 3.3. Sub-networks Performance

**MCM:** The value of the objective function *l*(γ) successfully converges to a value of 0.58 rad after around 170 iterations to obtain the values for the network parameters in Table 2. This allows to lower the mean value of the maximum deviation error from around 62 mm, following the tuning method introduced in Zahra et al. (2021c) to 46 mm in the studied workspace and reduction of the number of neurons per neuron assembly from 136 to 20 neurons. It can be noticed in Figure 12 that spikes occur in the fitness values, which indicates the balance held between exploration and exploitation while searching for the optimal values.

**Table 2:** Adaptive form of tree Parzen estimator (ATPE) tuning network parameters.

**Neuron parameters**							
	***a***	***b***	***c***	***d***	**A**_***s***_	**A**_***m***_	N
$l^{\dot{\theta_{i}}}$	0.07	-0.12	-68	6.8	5	0	20
$l^{\theta_{i}}$	0.22	0.15	-55	7.5	56	72	20
$l^{v_{j}}$	0.22	0.15	-55	7.5	56	72	20
**Synaptic connections**							
	***W***	**τ_*a*_**	**τ_*b*_**	***C*_*I*_**	***C*_*E*_**	*Itr* ^*^	
A2A	0.03	18	12	−4	4	4000	

**Figure 9:**
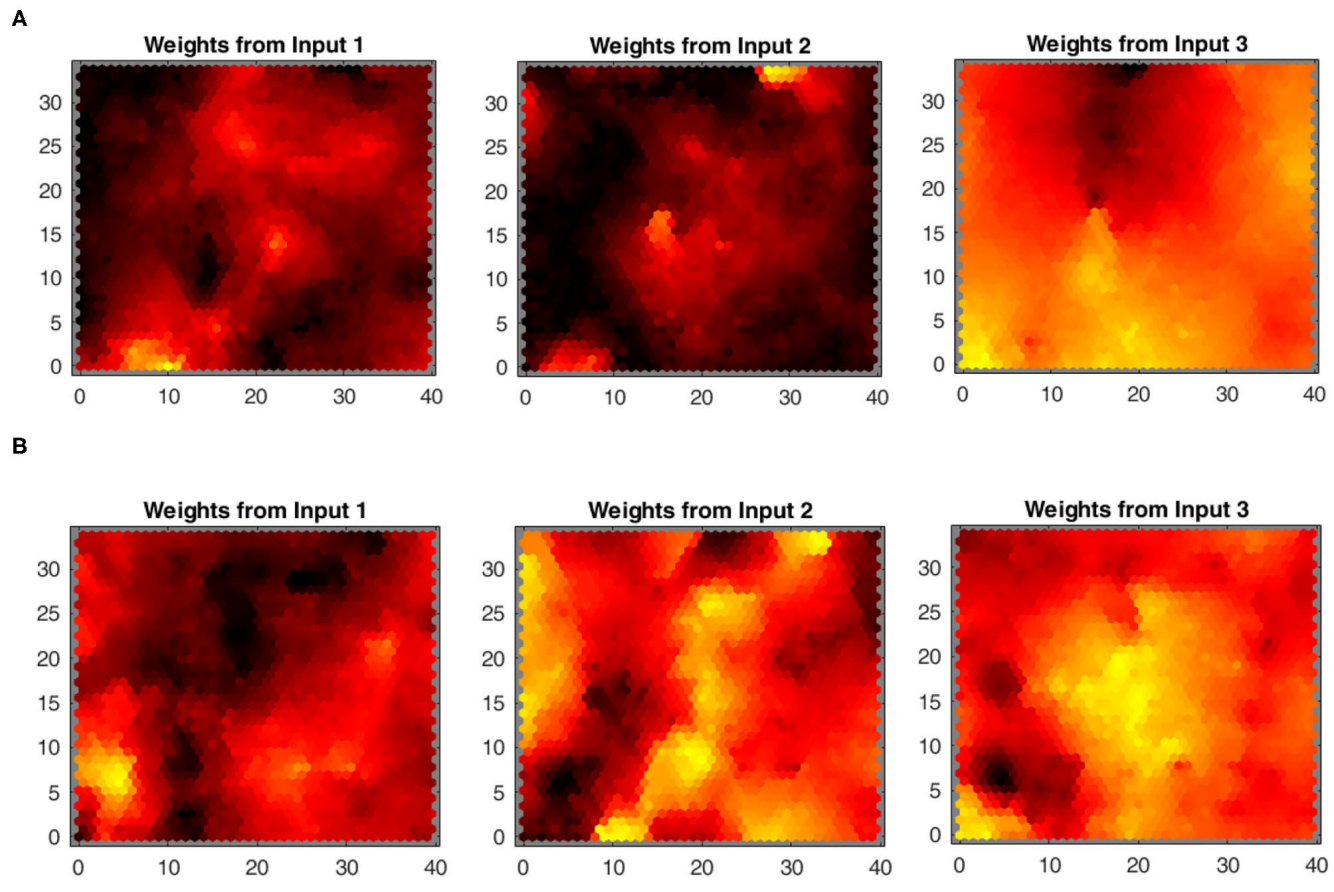
Heatmaps of the standard Kohonen SOM depicting the relation between each input from the joint spaces of **(A)** the human arm and **(B)** robot agents.

**Figure 10:**
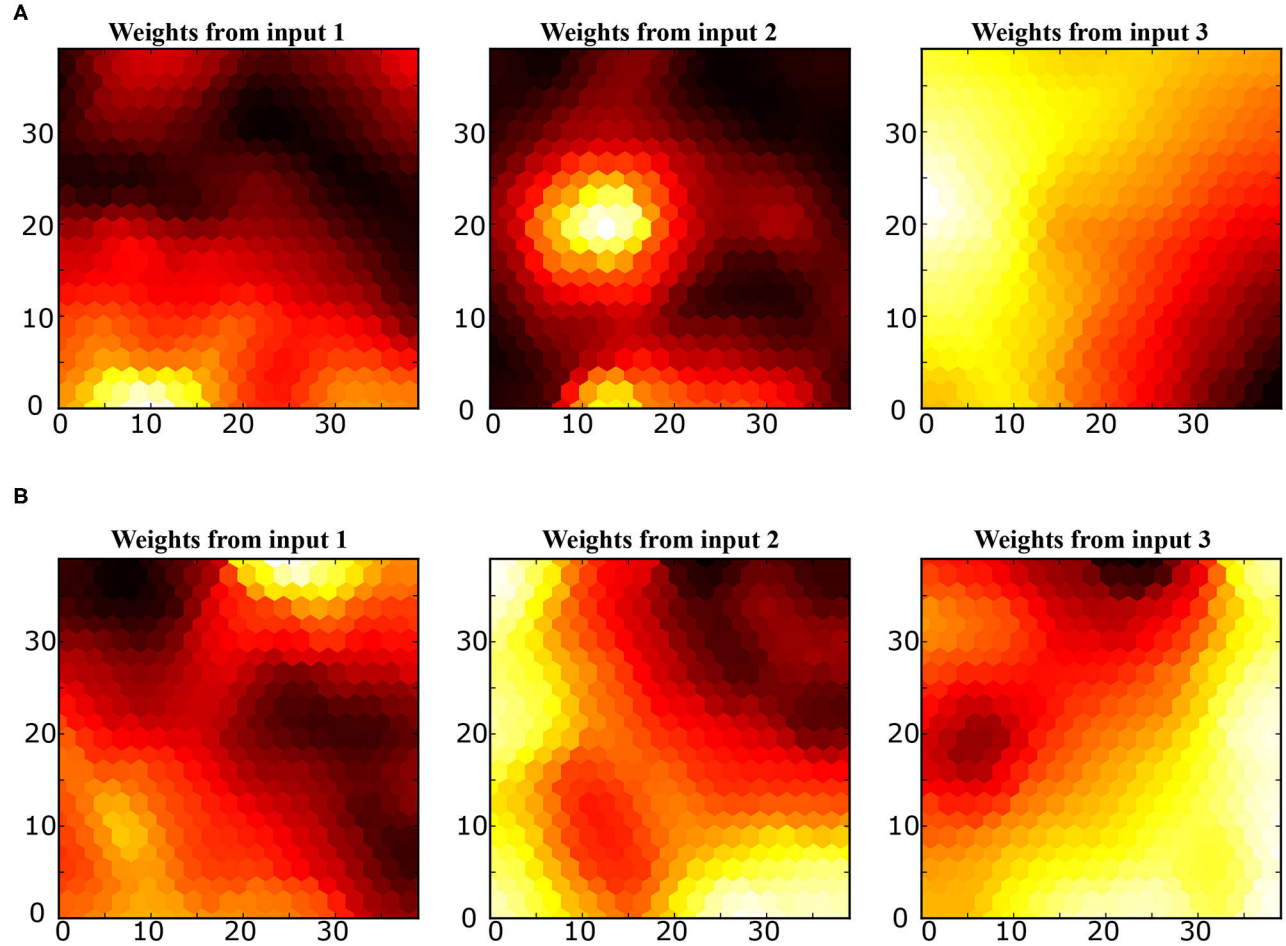
Heatmaps of the varying density SOM depicting the relation between each input from the joint spaces of **(A)** the human arm and **(B)** robot agents.

**SOM:** The mapping of the joint-spaces is studied first using the basic SOM developed by Kohonen, as shown in Figure 9, to provide a reference value for the improvement in the accuracy of the provided estimations for using the varying density SOM instead, as shown in Figure 10. A smooth gradient can be observed across the heatmaps in Figure 10 compared to Figure 9, which indicates a more uniform and even mapping in the case of the varying density SOM. The mean error in estimation is concluded to be approximately 0.25 and 0.16 rad in the case of the SOM compared to 0.17 and 0.11 rad in the case of varying density SOM for the human and robot agents, respectively. This allows for better estimation of the angular positions and, hence, angular velocities which improve the quality of the training data fed to the MCM.

### 3.4. Target Reaching

**Figure 11:**
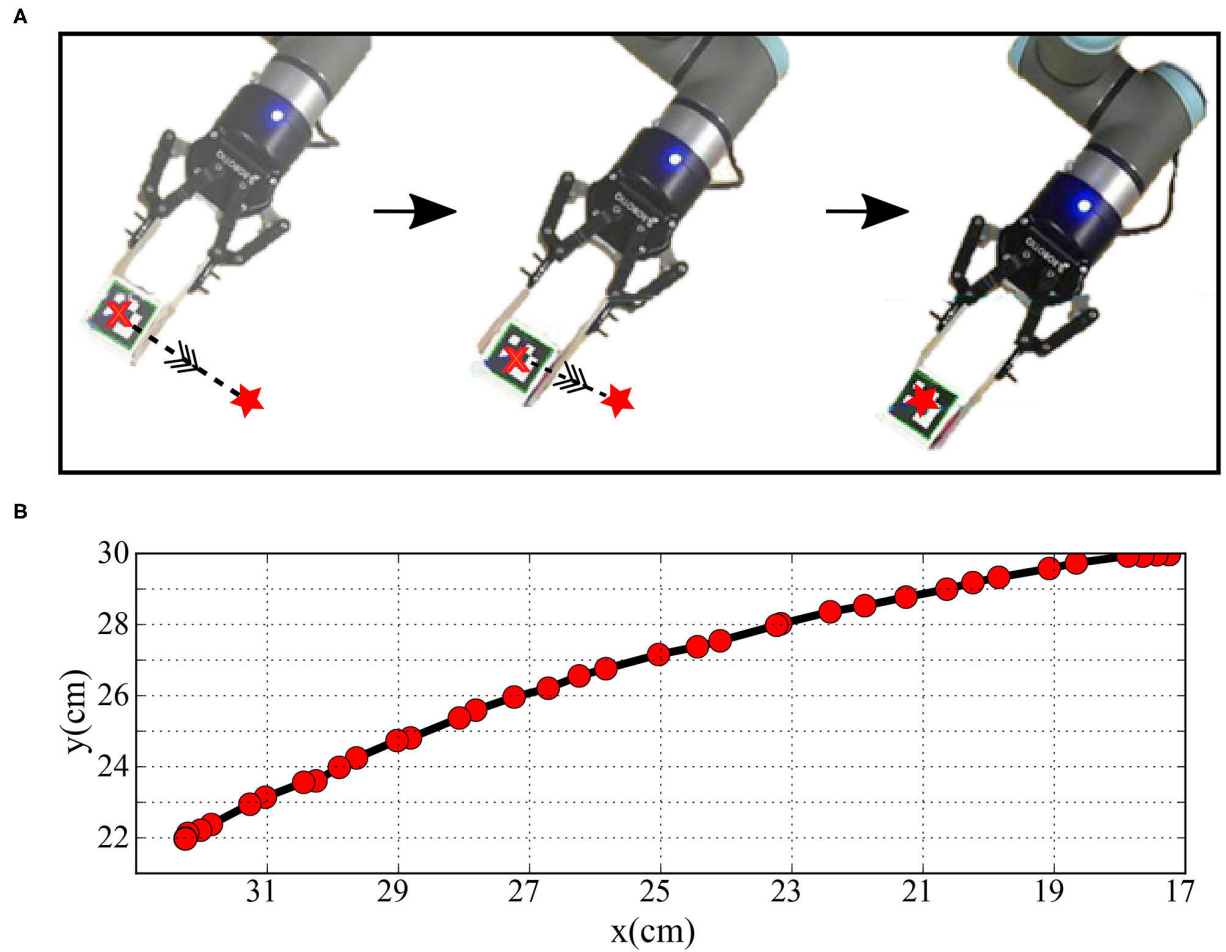
**(A)** The robot arm while moving after training by the transformed data and **(B)** the plot of the Cartesian position of the end effector while moving.

With the task to reach targets through a straight line as the shortest path, the end effector moves from the current position to a target position, as shown in Figure 11. First, the data collected from motor babbling is assessed in terms of the mean and SD of the maximum deviation from a straight line. The obtained values for the robot reaching [i.e., reproducing results from Zahra et al. (2021c)] are 4.2 and 2.3 cm for the mean and SD values, respectively, are bigger than those achieved by the human agent while recording the straight line reaching demonstrations with a mean and SD values of 2.1 and 1.3 cm, respectively. The teaching imitation data is then generated by introducing these examples to the *AJ-SOM* and recording the output from *RJ-SOM*. The mean and SD calculated for these examples to be equal to 3.4 and 1.9 cm, respectively, which proves the efficiency of the proposed network and the feasibility of improvement by the generated data.

Different percentages of contribution from the two sets of examples are employed to quantify the enhancement in the reaching movements in each case. Percentages of 30, 50, and 70 are applied with the quality of reaching movements recorded in each case and the results are obtained as shown in Table 3.

**Table 3:** Reaching results.

**Maximum deviation**		
	***Mean* (*mm*)**	***SD* (*mm*)**
30%	32.7	13.8
50%	28.1	10.9
70%	37.3	15.2

## 4. Discussion and Conclusion

In this study, the representation capabilities of the SOM and MCM are matched together to allow the robot to reduce the error while reaching targets. The static mapping of spaces by the SOM and the Oja-Hebbian synapses allow transforming human demonstrations into teaching examples in the robot's joint space. The MCM is trained by examples provided by motor babbling as well as demonstration examples to give the desired results.

**Figure 12:**
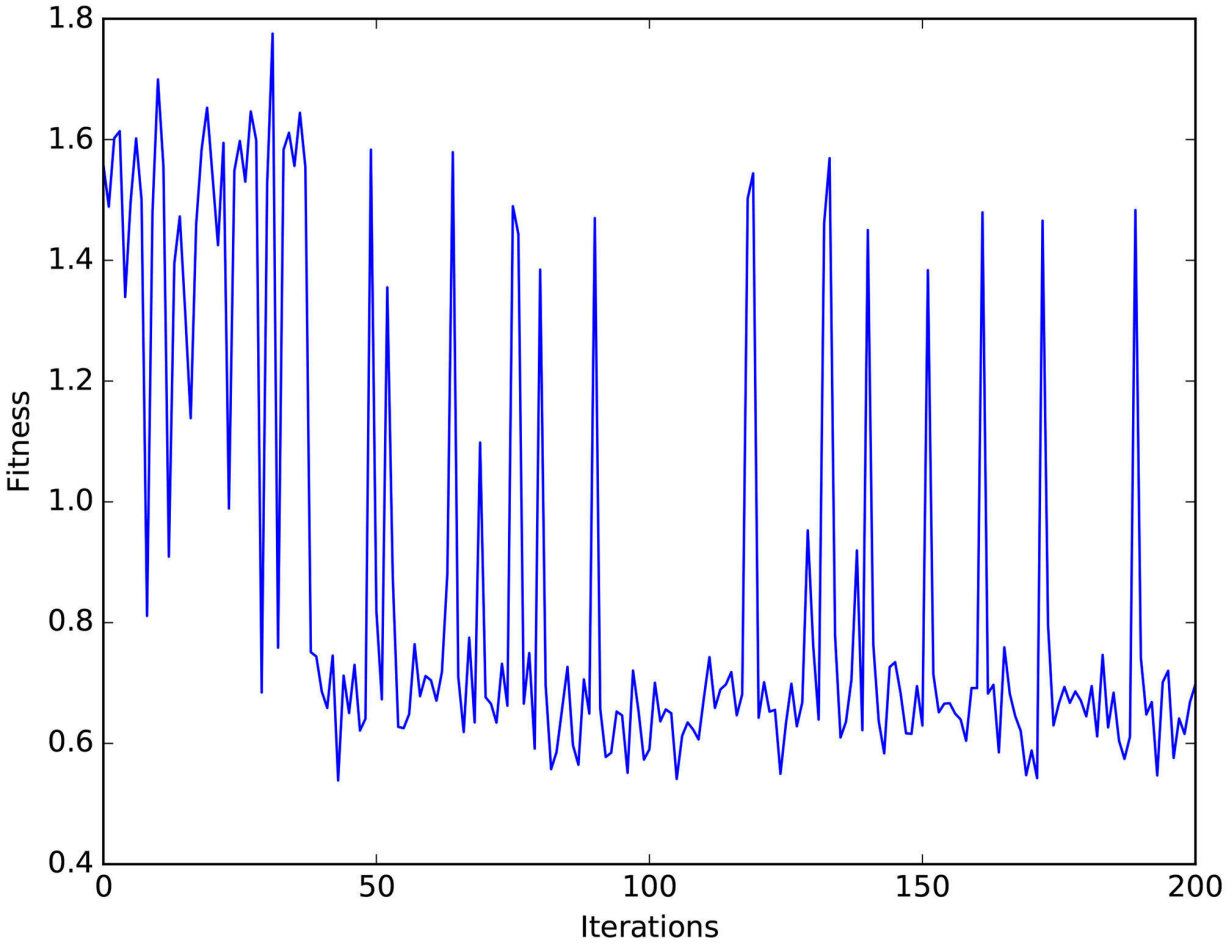
Values of the fitness function vs. the number of optimization iterations.

Using the varying density SOM reduces the error in static transformation compared to the basic SOM. Additionally, optimizing the parameters, as shown in Figure 12 and Table 2, of the MCM facilitates decreasing the error in the mapping and reducing the number of neurons in the network compared to relevant previous studies (Zahra et al., 2021c). The proposed method successfully decreases the deviation of the manipulator from the target path: first by applying Bayesian optimization introducing an improvement of around 25% and the post-optimization deviation is further reduced by 33% through imitation learning. It can be concluded as well that maintaining a good balance of self-generated data and “others” demonstration data helps obtain better results as shown in Table 3. Compared to Tieck et al. (2017) which utilizes an SNN to imitate grasping actions, the proposed system incorporates a solution for the correspondence issue and attains less error for a wider set of examples.

The proposed system does not take into account handling redundant solutions which shall be considered in future studies. Additionally, the equations ruling the amount and ratio of data from each of these categories shall be further investigated. A spiking model of the SOM shall be employed with a proper optimization technique as well, which would allow utilizing the incorporated temporal domain for faster learning, more biological plausibility, and energy efficient emulation while running in neuromorphic hardware (Evans and Stringer, 2012; Rumbell et al., 2013; Hazan et al., 2018; Khacef et al., 2020). Moreover, combining the cerebellar model with the developed network shall improve the performance and provide a good basis for a highly adaptive neural controller (Tolu et al., 2020; Zahra et al., 2021a,b).

## Data Availability Statement

The raw data supporting the conclusions of this article will be made available by the authors, without undue reservation.

## Author Contributions

OZ carried out the experiments under the supervision of DN-A and ST. PZ and AD helped structure the experiments and write the manuscript. All the authors discussed the results and contributed to the final manuscript.

## Funding

This work is supported in part by the Key-Area Research and Development Program of Guangdong Province 2020 under grant 2020B090928001, in part by the Research Grants Council of Hong Kong under grants 14203917 and 15212721, in part by the Jiangsu Industrial Technology Research Institute Collaborative Research Program Scheme under grant ZG9V, and in part by The Hong Kong Polytechnic University under grant UAKU.

## Conflict of Interest

The authors declare that the research was conducted in the absence of any commercial or financial relationships that could be construed as a potential conflict of interest.

## Publisher's Note

All claims expressed in this article are solely those of the authors and do not necessarily represent those of their affiliated organizations, or those of the publisher, the editors and the reviewers. Any product that may be evaluated in this article, or claim that may be made by its manufacturer, is not guaranteed or endorsed by the publisher.
